# Effects of Graphene Oxide and Reduced Graphene Oxide Nanostructures on CD4^+^ Th2 Lymphocytes

**DOI:** 10.3390/ijms231810625

**Published:** 2022-09-13

**Authors:** María José Feito, Mónica Cicuéndez, Laura Casarrubios, Rosalía Diez-Orejas, Sara Fateixa, Daniela Silva, Nathalie Barroca, Paula A. A. P. Marques, María Teresa Portolés

**Affiliations:** 1 Departamento de Bioquímica y Biología Molecular, Facultad de Ciencias Químicas, Universidad Complutense de Madrid, Instituto de Investigación Sanitaria del Hospital Clínico San Carlos (IdISSC), 28040 Madrid, Spain; 2 Departamento de Química en Ciencias Farmaceúticas, Facultad de Farmacia, Universidad Complutense de Madrid, Instituto de Investigación Sanitaria del Hospital Clínico San Carlos (IdISSC), 28040 Madrid, Spain; 3 Departamento de Microbiología y Parasitología, Facultad de Farmacia, Universidad Complutense de Madrid, Instituto de Investigación Sanitaria del Hospital Clínico San Carlos (IdISSC), 28040 Madrid, Spain; 4 Department of Chemistry and CICECO, University of Aveiro, 3810-193 Aveiro, Portugal; 5 Centre for Mechanical Technology & Automation (TEMA), Department of Mechanical Engineering, University of Aveiro, 3810-193 Aveiro, Portugal; 6 LASI—Intelligent Systems Associate Laboratory, 4804-533 Guimaräes, Portugal; 7 CIBER de Bioingeniería, Biomateriales y Nanomedicina, CIBER-BBN, 28040 Madrid, Spain

**Keywords:** graphene oxide, reduced graphene oxide, lymphocyte, CD4, CD3, immune response, cytokine

## Abstract

The activation of T helper (Th) lymphocytes is necessary for the adaptive immune response as they contribute to the stimulation of B cells (for the secretion of antibodies) and macrophages (for phagocytosis and destruction of pathogens) and are necessary for cytotoxic T-cell activation to kill infected target cells. For these issues, Th lymphocytes must be converted into Th effector cells after their stimulation through their surface receptors TCR/CD3 (by binding to peptide-major histocompatibility complex localized on antigen-presenting cells) and the CD4 co-receptor. After stimulation, Th cells proliferate and differentiate into subpopulations, like Th1, Th2 or Th17, with different functions during the adaptative immune response. Due to the central role of the activation of Th lymphocytes for an accurate adaptative immune response and considering recent preclinical advances in the use of nanomaterials to enhance T-cell therapy, we evaluated in vitro the effects of graphene oxide (GO) and two types of reduced GO (rGO15 and rGO30) nanostructures on the Th2 lymphocyte cell line SR.D10. This cell line offers the possibility of studying their activation threshold by employing soluble antibodies against TCR/CD3 and against CD4, as well as the simultaneous activation of these two receptors. In the present study, the effects of GO, rGO15 and rGO30 on the activation/proliferation rate of these Th2 lymphocytes have been analyzed by studying cell viability, cell cycle phases, intracellular content of reactive oxygen species (ROS) and cytokine secretion. High lymphocyte viability values were obtained after treatment with these nanostructures, as well as increased proliferation in the presence of rGOs. Moreover, rGO15 treatment decreased the intracellular ROS content of Th2 cells in all stimulated conditions. The analysis of these parameters showed that the presence of these GO and rGO nanostructures did not alter the response of Th2 lymphocytes.

## 1. Introduction

Due to its electrical conductivity, mechanical strength, topographical characteristics and high surface area, graphene is an excellent nanomaterial for developing scaffolds for tissue engineering (TE) [1]. Graphene oxide (GO) is more readily dispersible due to oxygen-containing functional groups on its surface [2]. It is renowned for its simple processability, high affinity for specific biomolecules and optimal cell-interface interactions [3]. For example, in neural TE, without the use of chemical inducers, GO-based substrates can support the adhesion, proliferation and differentiation of neurons [4]. Thermal, chemical and electrochemical treatments can convert non-conductive GO into rGO, which is conductive. Thermal treatment offers the benefit of avoiding the introduction of chemical moieties that can modify surface chemistry and exhibit intrinsic toxicity. Prior to employing these nanomaterials in TE; however, it is crucial to assess their safety. In this context, it is particularly important to study the interactions of nanomaterials with immune cells to determine their potential immunotoxicity [5].

The immune response comprises innate and adaptive defense mechanisms, which activate different cell populations in a coordinated manner. The innate immune response is the first line of defense against both infectious pathogens and foreign particles. This first response includes the recruitment of neutrophils, monocytes, dendritic cells and macrophages to sites of infection or biomaterial implantation, which carry out phagocytosis and produce antimicrobial peptides, reactive oxygen and nitrogen species (ROS and RNS, respectively) and chemical mediators (cytokines) [6]. Nanoparticles are phagocytosed and internalized by macrophages and dendritic cells that, acting as antigen-presenting cells (APCs), present foreign antigens to naïve T lymphocytes leading to their activation. Consequently, the stimulated T-cells secrete effector cytokines that modulate the immune response. Recent studies with nanoparticles are focused on the acute and/or chronic inflammatory response as well as on the possible modulation of these processes [7,8]. On the other hand, after contact of nanomaterials with biological fluids, different plasma proteins and biomolecules are adsorbed on their surface, modifying their physicochemical properties and determining their recognition and possible elimination by the cells of the innate immune system [9,10].

APCs serve to link the innate immune response to the antigen-specific adaptive response. These cells exhibit pattern-recognition receptors (PRR) on their surface, which in the case of the innate immune system mainly belong to the Toll-like receptors (TLR) family, which detect invading pathogens or signs of cell stress and damage. Cell activation mediated by TLR induces an inflammatory response through the NF-κB pathway, promoting the release of proinflammatory cytokines such as IL-1, IL-8, TNF-α and IL-12 [11]. In addition, the activation of TLR signaling pathways results in APC maturation, thus increasing the surface expression of co-stimulatory molecules on these cells, namely, CD80, CD86 and CD40, as well as increasing the surface expression of the Major Histocompatibility Complex (MHC type II) molecules. These surface markers on APCs are necessary for the adequate activation of T lymphocytes [12]. Mature APCs (carrying foreign peptides) migrate to secondary lymphoid organs, such as lymph nodes, where they act as professional APCs stimulating naïve T-cells.

Two types of T lymphocytes are established depending on the presence of the co-receptor molecules: helper T-cells with CD4^+^ phenotype and cytotoxic T-cells with CD8^+^ phenotype. CD4^+^ T-cells are mainly cytokine-secreting cells and can also assist antibody-producing B cells [13], while the CD8^+^ T-cells kill infected targets [14]. The expression of these markers is correlated not only with the specific effector function but also with the role in determining which class of MHC molecule is being recognized. CD4 and CD8 bind to MHC class II and MHC class I, respectively [15,16].

The activation of the virgin T lymphocytes occurs due to the interaction of their receptor and co-receptor molecules with the ligands present in the mature professional APCs (carriers of the antigenic peptides) [11,17]. Thus, a naïve T-cell will respond to the antigen only when an APC presents the specific antigen associated with MHC molecules to the T-cell receptor (TCR/CD3), but it is also necessary for the co-stimulatory signal through the B7 family (CD80, CD86) molecules in the APC which interact with CD28 on the surface of naïve T-cell [18]. Signaling through antigen-specific associated with MHC molecules presentation to the T-cell receptor TCR/CD3 is assisted by CD4 or CD8 co-receptors on the T cell, stabilizing TCR-mediated interactions and promoting intracellular communication. Concomitantly binding the different cytokines secreted by the APCs to specific receptors on the surface of the T lymphocyte causes differentiation in different subpopulations of effector cells with different functions, which allows great plasticity in the response of T lymphocytes.

In the case of helper T cells (with CD4^+^ phenotype), conversion to Th1 type cells can be promoted by IL-12 and IFN-Υ secreted by the APCs. Th1 helper cells generate immune responses against intracellular parasites, including bacteria and viruses. Conversely, Th2 cells generate immune responses against extracellular parasites, including helminths, and the presence of IL-4 and IL-10 promotes it. Finally, the subpopulation of Th17 cells is generated in the presence of IL-6 and IL-21, and they play a role in host defense against extracellular pathogens, particularly at the mucosal and epithelial barriers [19,20]. Each of these cells carries out specific immune responses associated with the affected tissue.

Another subpopulation of CD4^+^ T lymphocytes is the regulatory T-cells (Treg) that constitutively express high concentrations of the CD25 receptor and are primarily involved in suppressing autoreactive effector T-cells [19,20]. Recently, a subpopulation of CD4^+^ T-cells present in B-cell follicles, called follicular T helper (Tfh) cells, specialized in collaborating with B lymphocytes to trigger an adaptive immune response by producing specific antibodies, has been described [21]. Tfh cells express the chemokine receptor CXCR5, Bcl6, PD-1, ”inducible T-cell costimulator” (ICOS) and CD40L and release interleukin 21 [17]. In a similar manner, APCs also interact with cytotoxic CD8^+^ T-cells to promote their activation in order to perform their antigen-specific effector lytic function.

Due to the importance of T lymphocytes for the activation of the immune response and taking into account recent preclinical advances in the use of nanomaterials to enhance T-cell therapy [22], we have evaluated in vitro the effects of graphene oxide (GO) and two types of reduced GO (rGO15 and rGO30, obtained after vacuum-assisted heat treatment for 15 and 30 min, respectively) nanostructures, on the CD4^+^ Th2 lymphocyte cell line SR.D10. The present study includes the culture of SR.D10 lymphocytes and their stimulation with soluble antibodies against TCR/CD3 and against CD4, as well as the simultaneous activation of these two receptors. The effects of GO, rGO15 and rGO30 on the proliferation of these Th2 lymphocytes and their correct activation were analyzed by studying cell viability, cell cycle phase, intracellular content of reactive oxygen species (ROS) and cytokine secretion, which have been extensively implicated in T-cell response and indicate the activation state of the lymphocyte.

## 2. Results and Discussion

### 2.1. Structural and Morphological Analysis of GO, rGO15 and rGO30 Nanostructures

Graphene oxide (GO) and two types of reduced graphene oxide (rGO15 and rGO30) thermally reduced at 200 °C were characterised using vibrational spectroscopies, particularly infrared and Raman spectroscopies, to complement the characterisation of these nanostructures provided in our previous paper [23]. The zeta (ζ) potentials of GO and rGO aqueous dispersions at pH 6.8 were determined. This pH value was obtained after the dispersion of the samples in distilled water without adjustments. As the objective was to compare their surface charge, no pH adjustments were necessary. The values were −35.2 ± 2.7 mV for GO; −32.3 ± 2.5 mV for GO15 and −26.6 ± 2.1 mV for rGO30 confirming the high negative charge of GO and the mild reduction degree, particularly of GO15.

**Figure 1:**
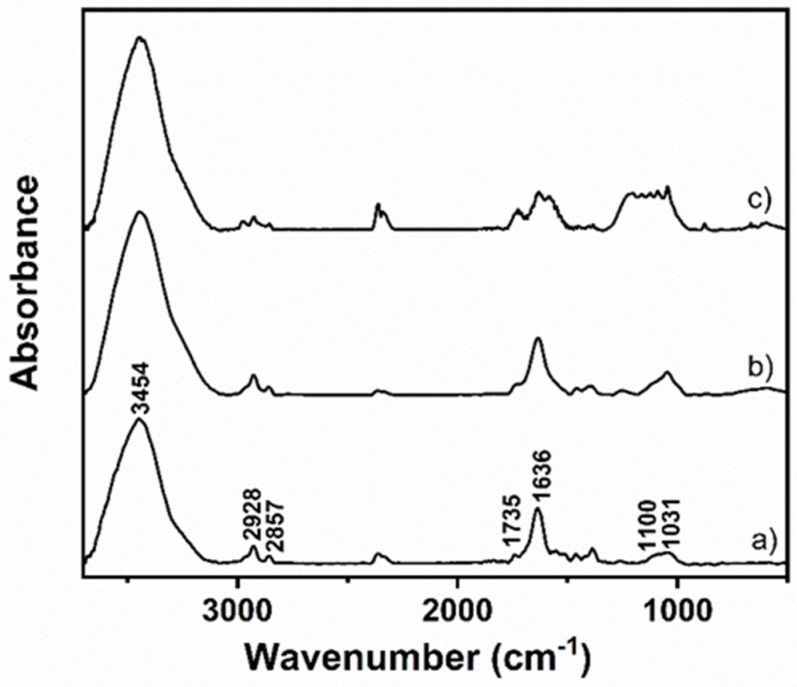
FTIR spectra of GO (**a**), rGO15 (**b**) and rGO30 (**c**).

Figure 1 shows the FTIR spectra of the three samples. The vibrational bands of GO are assigned in accord with the literature [24,25,26,27]: υ(O-H) broad band from water at 3454 cm^−1^, υ(C=O) from carbonyl and carboxylic groups at 1735 cm^−1^, H–O–H bending vibration of water molecules and the skeletal C=C bond vibrations of the graphitic domains at 1636 cm^−1^, υ(C-OH) at 1100 cm^−1^, υ(C-O) of the epoxy group at 1031 cm^−1^. Additionally, the bands at 2928 and 2857 cm^−1^ assigned to methylene stretch (CH/CH_2_) indicate the existence of some CH_2_ or CH groups in the GO samples.

In the case of the rGO samples, rGO15 did not differ significantly from GO, indicating that 15 min of thermal treatment is insufficient to generate major changes in the FTIR spectrum. However, the rGO30 exhibits differences in its FTIR spectrum. The observed changes include the splitting of the GO band at 1636 cm^−1^, with the appearance of a new band at 1575 cm^−1^, which can be assigned to the C=C stretching [28]. This can be justified by the release of water molecules, thus lowering the H-O-H contribution, together with the restoration of graphene domains during the heating process, which suggests more sp^2^ bonds in rGO30 compared to GO and rGO15. The broad band between 1215 and 1041 cm^−1^ is primarily attributed to C-OH groups [24]. These findings are supported by ζ values measured for these samples. Although there is an effective reduction of GO after 30 min of thermal treatment, as supported by the C1s XPS data in our previous article showing the clear decrease in the intensity of the carbon peaks associated with C-O groups accompanied by an increase in the C-C bonds after reduction [23], there are still a significant number of oxygen functional groups on the nanosheets’ surface, confirmed by the low ζ value of −26 mV. Bringing up that the induced exfoliation that occurs during the heating process as a result of the release of water vapour, CO_2_ and CO increase the exposed surface area of the reduced samples may help to explain why the FTIR bands for the rGO30 sample present a higher resolution for the peaks in this area of the spectrum.

**Figure 2:**
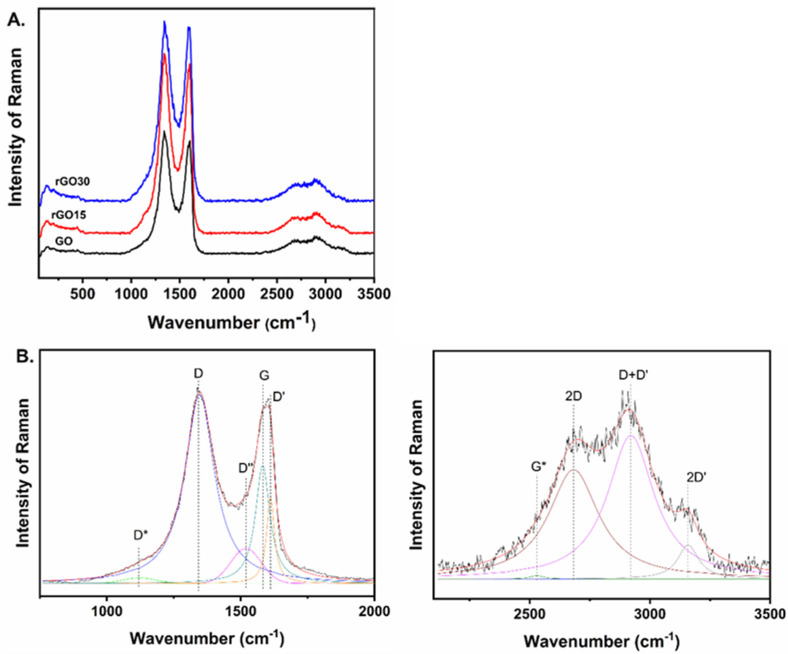
(**A**) Raman spectra of GO and reduced GO (rGO15 and rGO30). (**B**) Deconvolution of the Raman spectrum of GO.

The Raman spectra of the GO and reduced GO (rGO15 and rGO30) deposited on a glass slide were recorded and presented in Figure 2A. All three Raman spectra present D and G bands centred at ~1345 and ~1585 cm^−1^, respectively and the splitting of a second-order band (2D) at 2900 cm^−1^ [29,30]. No significant differences can be observed between the Raman spectra prior to deconvolution.

The G band is assigned to the first-order E_2g_ optical mode of graphite and the in-plane stretching of the C=C bonds. The D band is related to an A_1g_ breathing mode at the Brillouin zone boundary, and its intensity is usually associated with the number of defects in the graphene plane [31]. The Raman features analysis of graphene derivatives such as GO and rGO often neglected additional bands or shoulders appearing in the Raman spectra between 1100 and 1800 cm^−1^. The first-order bands in the Raman spectra were fitted using five functions (two Gauss and three Pseudo-Voigt), which were ascribed as D, G, D*, D’ and D” [29,32]. An illustrative example for GO is presented in Figure 2B-left. Similar analyses were performed for the reduced-GO materials and are presented in SI, Figure S1.

The second-order bands are detected in the range from 2500 to 3200 cm^−1^. The 2D band (also called G’) originates from a double-resonance intervalley Raman scattering process with the two iTO photons at the K point [31]. The second-order bands were fitted into four Lorentzian functions (G*, 2D, D+D’, 2D’), and an illustrative example for GO is presented in Figure 2B-right. Similar analyses for the rGO samples are shown in SI (Figure S1). Tables S1 and S2 of the SI show the band parameters of the fits and the ratios between the specific bands.

It has been reported that as the oxygen content of rGO decreases, the band position of D* increases [32]. This band is related to disordered graphitic lattices provided by sp^2^−sp^3^ bonds. Our results agree that a shift of the D* band position towards higher wavenumber is observed with the increase of heat treatment’s time, suggesting a reduction of the oxygen groups in the GO samples (Table S1). In addition, the effects of reduction on the Raman spectra for laser-reduced GO have also been reported by Zhang et al. [33]. They reported an increase in the D/G intensity ratio for the femtosecond laser reduction of GO. We have calculated the I_D_/I_G_ ratio for GO and rGO (rGO15 and rGO30) (Table S2), and an increase in the I_D_/I_G_ ratio values was also observed for reduced GO.

Another indication of the reduction of the rGO samples (rGO15 and rGO30) is the decrease of the area ratio A_D+D’_/A_D_, A_2D’_/A_D_ and A_2D’_/A_D+D’_ as the time of the heat treatment increase, and also the degree of reduction increases, which is more evident for rGO30. This behaviour has also been demonstrated for laser-irradiated rGO samples [30].

Although the value obtained for the ID/IG ratio for rGO15 is higher than rGO30, the areas ratio A_D+D’_/A_D_, A_2D’_/A_D_ and A_2D’_/A_D+D’_ for rGO15 are very similar to the values obtained for GO. Overall, the results indicate that rGO30 was more reduced than rGO15 following heat treatment.

### 2.2. Effects of GO, rGO15 and rGO30 on the Intracellular Complexity of CD4^+^ Th2 SR.D10 Lymphocytes in Basal and Stimulated Conditions

The Th2 lymphocytes are specialized cells of the immune system which play a central role as mediators of the cellular response. Their activation influences other components of the immune system in different physiological contexts. SR.D10 cells “syngeneic-reactive D10” were isolated from the mouse CD4+ helper T-cell clone, D10.G4.1 (D10). It is a differentiated line of CD4+ Th2 lymphocytes that shows hyperreactivity to TCR-dependent and -independent stimuli, indicating it has a lower activation threshold than the original clone. These clones also offer the possibility of culturing them in the presence of an interleukin-rich medium, which makes this cell line an experimental model of great interest for analyzing mechanisms of T-cell activation [34]. Moreover, Th2 SR.D10 cell stimulation can be achieved through the TCR-CD3 and CD4 lymphocyte receptors when monoclonal antibodies YCD3-1 (anti-CD3) and GK1.5 (anti-CD4), respectively, are added to the cell culture (referred in the present work as stimulated conditions).

In order to know the possible activation effects of GO, rGO15 and rGO30 nanostructures on Th2 cells, the intracellular complexity of a differentiated Th2 SR.D10 lymphocyte line [34,35] was quantified by flow cytometry analyzing a 90° light scatter (side scatter, SSC) after 24 h of incubation. SSC is proportional to the intracellular complexity determined in part by the cellular cytoplasm, mitochondria and pinocytic vesicles [36]. For this reason, this parameter is employed as a measure of lymphocyte proliferation and activation [37]. In addition, SSC has also been used as an indicator of the incorporation of nanoparticles inside cells [38,39]. As a positive control for activation, the lymphocytes were cultured with interleukin-rich medium (+ILs medium).

**Figure 3:**
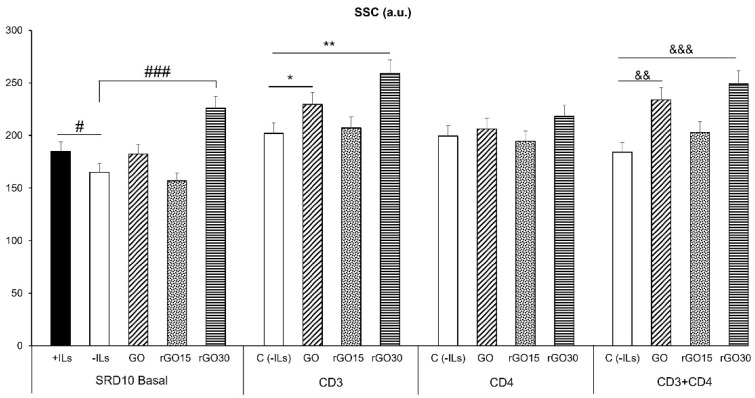
Effect of GO, rGO15 and rGO30 on the complexity of the CD4^+^ Th2 SR.D10 cell line after 24 h of treatment with 5 µg/mL of these nanostructures, evaluated by flow cytometry analyzing 90◦ light scatter (side scatter, SSC) under basal and activated conditions. Statistical significance: # *p* < 0.05 (compared to positive control cells without material cultured in the presence of ILs); ### *p* < 0.005 (compared to control cells without material cultured in the absence of ILs); * *p* < 0.05, ** *p* < 0.01 (comparison between nanostructures to control activated through CD3); ^&&^ *p* < 0.01, ^&&&^ *p* < 0.005 (comparison between nanostructures to control activated through CD3 and CD4).

Figure 3 shows a significant increase of the intracellular complexity (SSC) of Th2 SR.D10 lymphocytes cultured in the presence of a medium supplemented with interleukins (+ILs, black bar) compared to control SR.D10 lymphocytes in the absence of ILs (-ILs, white bar) in basal conditions, thus evidencing the correct activation of lymphocytes by the presence of ILs. Regarding the results obtained with GO, rGO15 and rGO30 nanostructures in basal conditions, we only observed a significant SSC increase (*p* < 0.005) in the presence of rGO30. A similar complexity increase was obtained in previous studies with another representative cell line of the innate immune response—the RAW 264.7 macrophages—after exposure of these cells to mesoporous bioactive glass nanoparticles (nanoMBGs) due to the intracellular nanoMBG incorporation [40]. In contrast, no changes were observed in the complexity of lymphocytes after the treatment with GO and rGO15. In this case, as we previously observed in studies with MC3T3-E1 preosteoblasts, the incorporation of nanoMBGs did not affect the complexity of preosteoblasts [41]. These differences could be due to the fact that, as is well known, the incorporation and effects of nanoparticles depend on their characteristics and cell type [42].

The study was further completed by analyzing the effects of GO, rGO15 and rGO30 nanostructures on cell complexity of Th2 SR.D10 lymphocytes under stimulated conditions by specific activation of the TCR/CD3 receptor (antigen T-cell receptor), CD4 surface receptor (main co-receptor of T helper cells), and by co-stimulating through TCR-CD3 and CD4. To carry out the activation assays, cultured cells were thoroughly washed to remove interleukins present in the culture medium and were then stimulated with anti-CD3, anti-CD4 or anti CD3+CD4 monoclonal antibodies for 24 h. As shown in Figure 3, higher SSC values were obtained under all stimulated conditions than under basal conditions, in the absence and the presence of GO, rGO15 and rGO30, demonstrating the efficient use of the SSC as a measure of cell activation [37]. These results indicate that lymphocytes respond adequately to all stimuli, and the presence of these nanostructures did not significantly change the conditions of lymphocyte activation. A significant increase was observed under stimulated conditions with CD3 and CD3+CD4 in the presence of GO and rGO30 compared to the corresponding control in the absence of nanomaterial.

### 2.3. Proliferation of CD4^+^ Th2 SR.D10 Lymphocytes after GO, rGO15 and rGO30 Treatment in Basal and Stimulated Conditions

In the present study, we evaluated in vitro the mitochondrial activity of Th2 SR.D10 lymphocytes to measure their survival and proliferation after GO, rGO15 and rGO30 treatment in basal conditions and to follow specific activation of the TCR/CD3 receptor, CD4 surface receptor and by co-stimulating through TCR/CD3 and CD4. Activation of lymphocytes promotes differentiation processes that will determine the type of cell they will become and the specific cytokines they will secrete. To carry out the activation assays, cultured cells were washed extensively to remove interleukins present in the medium and then subjected to stimulation with the above-mentioned antibodies.

**Figure 4:**
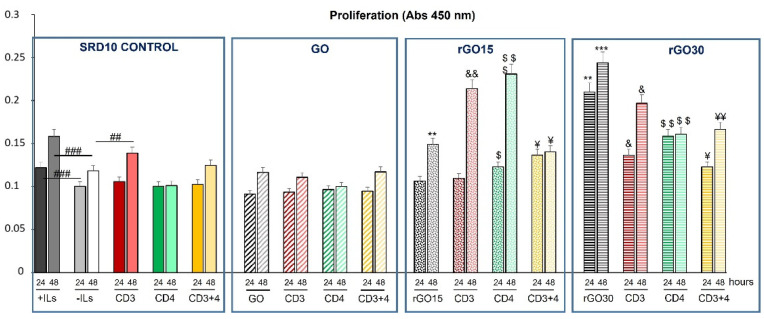
Effect of GO, rGO15 and rGO30 on the proliferation of CD4^+^ Th2 SR.D10 cell line after 24 h or 48 h of treatment with 5 µg/mL of these nanostructures, evaluated by mitochondrial activity assays under basal and stimulated conditions. Statistical significance: ### *p* < 0.005 (compared to positive control cells without material cultured in the presence of ILs at 24 and 48 h), ## *p* < 0.01 (compared to control cells without material cultured in the absence of ILs activated through CD3); ** *p* < 0.01 (comparison between rGO15 and rGO30 to control); *** *p* < 0.005; ^&^ *p* < 0.05, ^&&^ *p* < 0.01 (compared to control cells with rGO15 and rGO30 in the absence of ILs activated through CD3); ^$^ *p*< 0.05, ^$$^ *p*< 0.01, ^$$$^ *p*< 0.005 (comparison between rGO15 and rGO30 to control activated through CD4); ^¥^ *p*< 0.05, ^¥¥^ *p*< 0.01 (comparison between rGO15 and rGO30 to control activated through CD3 and CD4).

Figure 4 shows the proliferation results obtained with Th2 SR.D10 lymphocytes stimulated for 24 and 48 h with anti-CD3, anti-CD4 or anti CD3+CD4 monoclonal antibodies in the absence or presence of GO, rGO15 and rGO30. In the figure, rectangles have been drawn to indicate four different situations from left to right: SRD10 CONTROL rectangle indicates control lymphocytes in the absence of material; GO rectangle indicates lymphocytes in the presence of GO; rGO15 rectangle indicates lymphocytes in the presence of rGO15; rGO30 rectangle indicates lymphocytes in the presence of rGO30. Within each rectangle, the bars corresponding to the values of the unstimulated lymphocytes are shown on the left (indicating where necessary the material present GO, rGO15, rGO30). On the right, within each rectangle, are the values of the lymphocytes treated with each type of stimulus (CD3, CD4, CD3+CD4) in each situation.

As expected, under basal conditions without stimuli, in the absence of interleukin-rich medium (-ILs bars), lymphocyte proliferation was lower than in the presence of interleukins (+ILs bars), as mentioned above. As can be observed (Figure 4), the lymphocyte proliferation was time-dependent and increased in all cases. The presence of GO did not modify cell proliferation; however, rGO15 and rGO30 significantly induced this process after 48 h of treatment in the absence of stimuli, highlighting the pronounced effect of rGO30. In the presence of stimuli, the increase of proliferation induced by rGO15 and rGO30 was maintained in all cases, although a slight but significant increase was observed in the case of rGO15 after stimulation through CD3 together with the co-receptor CD4 at 48 h. The pronounced effect of rGO30 masked the possible action of CD3 and CD4 stimulation.

### 2.4. Effects of GO, rGO15 and rGO30 on Cell-Cycle Phases of CD4^+^ Th2 SR.D10 Lymphocytes in Basal and Stimulated Conditions

**Figure 5:**
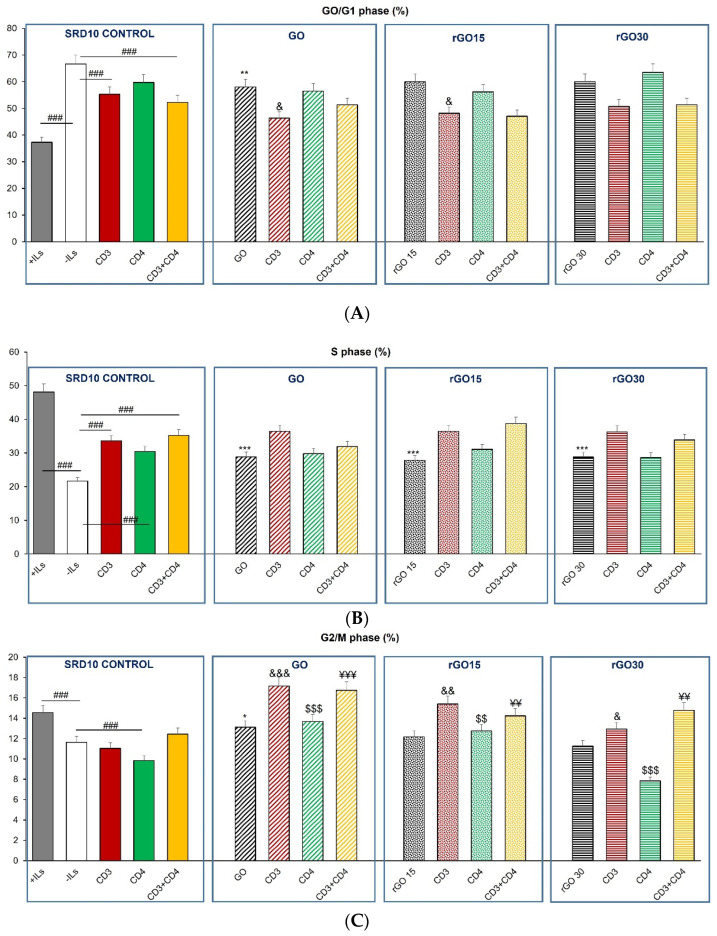
(**A**) Effect of GO, rGO15 and rGO30 on the percentage of G_0_/G_1_ phase of CD4^+^ Th2 SR.D10 cell line after 24 h of treatment with 5 µg/mL of these nanostructures, evaluated by flow cytometry under basal and stimulated conditions. The control cells were cultured in the presence of stimuli without material (SR.D10 CONTROL) in parallel. Statistical significance: ### *p* < 0.005 (compared to control cells without material cultured in the absence of ILs); ** *p* < 0.01 (compared to control cells without material cultured in the absence of ILs); ^&^ *p* < 0.05 (compared to control cells activated through CD3 in the absence of ILs). (**B**) Effect of GO, rGO15 and rGO30 on the percentage of the S phase of CD4^+^ Th2 SR.D10 cell line after 24 h of treatment with 5 µg/mL of these nanostructures, evaluated by flow cytometry under basal and stimulated conditions. The control cells were cultured in the presence of stimuli without material (SR.D10 CONTROL) in parallel. Statistical significance: ### *p* < 0.005 (compared to control cells without material cultured in the absence of ILs); *** *p* < 0.005 (compared to control cells without material cultured in the absence of ILs). (**C**) Effect of GO, rGO15 and rGO30 on the percentage of G_2_/M phase of CD4^+^ Th2 SR.D10 cell line after 24 h of treatment with 5 µg/mL of these nanostructures, evaluated by flow cytometry under basal and stimulated conditions. The control cells were cultured in the presence of stimuli without material (SR.D10 CONTROL) in parallel. Statistical significance: ### *p* < 0.005 (compared to control cells without material cultured in the absence of ILs); * *p* < 0.05 (compared to control cells without material cultured in the absence of ILs); ^&^ *p* < 0.05, ^&&^ *p* < 0.01, ^&&&^ *p* < 0.005 (compared to control cells activated through CD3 in the absence of ILs); ^$$^ *p*< 0.01, ^$$$^ *p*< 0.005 (compared to control cells activated through CD4 in the absence of ILs); ^¥¥^ *p*< 0.01, ^¥¥¥^ *p*< 0.005 (compared to control cells activated through CD3 and CD4 in the absence of ILs).

The cell-cycle phases (G_0_/G_1_, S and G_2_/M) of murine CD4^+^ Th2 SR.D10 lymphocytes were analyzed after 24 h of treatment with GO, rGO15 and rGO30 nanostructures under basal conditions and following specific activation of the TCR/CD3 receptor, CD4 surface receptor, and by co-stimulating through TCR/CD3 and CD4. The obtained results are represented in Figure 5A–C.

Figure 5A shows that in the absence of the material, the SR.D10 control cells cultured with an interleukin-rich culture medium (+ILs, gray bar) presented a lower percentage in the G_0_/G_1_ phase (*p* < 0.005) than in the absence of interleukins (-ILs, white bar), in agreement with the effect of ILs on the proliferation of this cell type [34]. On the other hand, the presence of the stimuli in the absence of ILs slightly decreased the G_0_/G_1_ percentage, according to the proliferation results obtained and shown in Figure 4. The presence of 5 μg/mL of GO, rGO15 and rGO30 did not modify the effects induced by the stimuli (CD3, CD4 and CD3/CD4) on the G_0_/G_1_ phase, obtaining the same type of profile in response to these stimuli as that observed in the absence of the material.

In S and G_2_/M phases (Figure 5B,C, respectively), a pronounced increase was observed in the control cells cultured in the presence of ILs (+ILs, gray bar) compared to the cells cultured in the absence of ILs (-ILs, white bar). These results are in agreement with the proliferative effect induced by the ILs present in the culture medium.

The presence of stimuli in the absence of ILs significantly increased the % S phase in SR.D10 control cells. The treatment with GO, rGO15 and rGO30 in the absence of stimuli increased the S-phase percentage with respect to the control, in agreement with the proliferative effect (Figure 5B). This tendency was maintained in the case of the G2/M phase (Figure 5C).

The presence of these materials in conditions stimulated by CD3 and CD4 did not alter the percentages observed in the absence of the material in S phase (Figure 5B). Therefore, the behavior of the CD4^+^ Th2 SR.D10 lymphocytes against the different stimuli presented the same pattern in the presence of these nanostructures.

Regarding the G_2_/M phase (Figure 5C), the percentages obtained in the absence of materials and without ILs were lower than the values obtained in the presence of ILs, demonstrating the importance of this interleukin-enriched medium in the proliferation of these cells (Click medium supplemented with 10% FCS, 5 U/mL of recombinant mouse mrIL-2, 10 U/mL of mrIL-4 and 25 pg/mL of mrIL-1).

In summary, it can be concluded that the analysis of the different phases of the cell cycle showed that the specific stimuli (CD3 and CD4) produced the appropriate CD4^+^ Th2 SR.D10 lymphocyte response that was maintained in the presence of GO and rGO.

### 2.5. Effects of GO, rGO15 and rGO30 on Cell viability and Intracellular Reactive Oxygen Species (ROS) Content of CD4^+^ Th2 SR.D10 Lymphocytes in Basal and Stimulated Conditions

In recent years, different experimental models have been used in vivo and in vitro to study the cytotoxicity induced by graphene and its derivatives. Numerous articles have been published on the oxidative stress induced by these materials and their interaction with immune response system components [23]. Overproduction of reactive oxygen species (ROS) can induce failure of cells to maintain their physiological function and result in an imbalance between their excessive generation and the limited antioxidant defense, leading to adverse biological effects, such as protein denaturation, membrane lipid peroxidation, mitochondrial and DNA changes [43]. Taking all these facts into account, in the present study, we have evaluated the cell viability and intracellular ROS content of CD4^+^ Th2 SR.D10 lymphocytes after 24 h of treatment with GO, rGO15 and rGO30 nanostructures under basal conditions and following specific activation of the TCR-CD3 receptor, CD4 surface receptor, and by co-stimulating through TCR/CD3 and CD4. The presence or absence of ILs in the culture medium and cell control conditions without nanomaterial were performed as described above.

**Figure 6:**
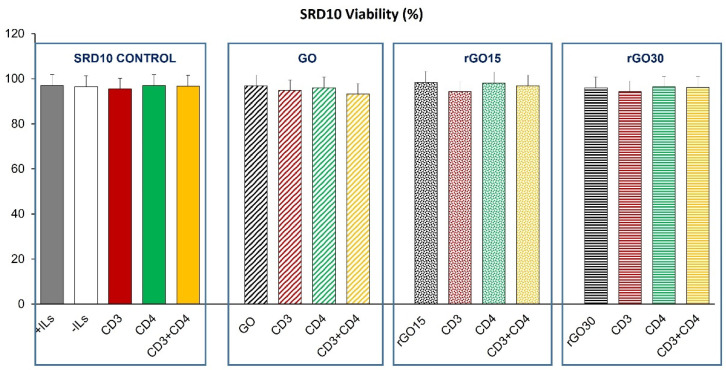
Effect of GO, rGO15 and rGO30 on the viability of CD4+ Th2 SR.D10 cell line after 24 h of treatment with 5 µg/mL of these nanostructures, evaluated by flow cytometry under basal and stimulated conditions. The control cells were cultured in the presence of stimuli without material (SR.D10 CONTROL) in parallel.

As can be observed in Figure 6, high viability values were obtained after treatment with these nanostructures in basal and stimulated conditions.

**Figure 7:**
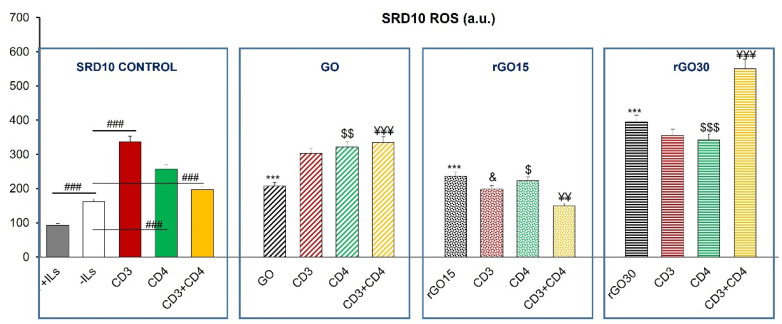
Effect of GO, rGO15 and rGO30 on the intracellular content of reactive oxygen species (ROS) of CD4^+^ Th2 SR.D10 cell line after 24 h of treatment with 5 µg/mL of these nanostructures, evaluated by flow cytometry under basal and stimulated conditions. The control cells were cultured in the presence of stimuli without material (SR.D10 CONTROL) in parallel. Statistical significance: ### *p* < 0.005 (compared to control cells without material cultured in the absence of ILs); *** *p* < 0.005 (compared to control cells without material cultured in the absence of ILs); ^&^ *p* < 0.05 (compared to control cells activated through CD3 in the absence of ILs); ^$^ *p* < 0.05, ^$$^ *p* < 0.01,^$$$^ *p* < 0.005 (compared to control cells activated through CD4 in the absence of ILs); ^¥¥^ *p* < 0.01, ^¥¥¥^ *p* < 0.005 (compared to the control cells activated through CD3 and CD4 in the absence of ILs).

Figure 7 shows the effect of GO, rGO15 and rGO30 on the intracellular ROS content of the CD4^+^ Th2 SR.D10 cell line. In the absence of these nanostructures, a ROS increase was observed after stimulation through the TCR/CD3 and CD4 lymphocyte receptors when monoclonal antibodies YCD3-1 (anti-CD3) and GK1.5 (anti-CD4), respectively, were added to SR.D10 cells, demonstrating the sensitivity of the assay. This effect was reversed when lymphocytes received the two stimuli at the same time (CD3+CD4), in agreement with their proliferative effect and with the significant increase observed in the percentage of the S phase (Figure 4 and Figure 5B, respectively).

The presence of GO, rGO15 and rGO30 produced in all cases a significant increase in the intracellular ROS content compared to the control cells without material cultured in the absence of ILs under the conditions studied. Treatment with rGO30 induced higher levels of ROS than treatment with GO and rGO15. However, a decrease in this parameter was observed with rGO15 under stimulation with CD3, CD4 and more pronounced with the combined treatment of CD3 together with CD4. Nevertheless, CD3 plus CD4 coactivation in the presence of rGO30 induced a pronounced increase in the production of ROS, which is likely due to a synergic effect of the two stimuli and the nanomaterial. On the other hand, the significant change in SSC previously observed only after the treatment with rGO30 (Figure 3) could be related to the higher levels of lymphocyte proliferation (Figure 4) and reactive oxygen species (ROS, Figure 7) induced by this material.

It should be noted that a possible protective effect of CD3 stimulation was observed in the presence of rGO15 and rGO30 but not with GO. These results indicate a beneficial role of GO reduction on this particular aspect of the lymphocyte response to a specific stimulus, in agreement with the beneficial effects due to this reduction process observed in previous studies with macrophages [23].

These assays allowed us to observe the correct functioning of all these stimuli and the beneficial effect of graphene reduction under conditions of lymphocyte stimulation, especially with rGO15, when the different types of stimulated conditions are considered in general terms. The fact that the ROS values obtained in the presence of rGO15 are lower than in the presence of rGO30 could also be related to the lower lymphocyte proliferation observed with rGO15 compared to rGO30 (Figure 4).

### 2.6. Effects of GO, rGO15 and rGO30 on Interleukin-4 (IL-4) secretion by CD4^+^ Th2 SR.D10 Lymphocytes in Basal and Stimulated Conditions

Although GO, rGO15 and rGO30 have not been observed to affect lymphocyte activation, their potential inflammatory effect should be evaluated. In this regard, it has recently been shown that mouse peritoneal macrophages polarized towards a M2 phenotype produce regulatory cytokines, including IL-4, IL-13, TGF-β and IL-10 that might suppress T-cell proliferation in vitro. Some cytokines, such as IL-4 and IL-10, are critical for proper tissue repair and regeneration due to their role in switching from M1 (proinflammatory) to M2 (anti-inflammatory) phenotype [44].

To study in depth the potential effect of these nanomaterials on the anti-inflammatory cytokine secretion, in the present work, we quantified the levels of IL-4 secreted by cultured Th2 SR.D10 lymphocytes after treatment with GO, rGO15 and rGO30 under basal and stimulated conditions.

**Figure 8:**
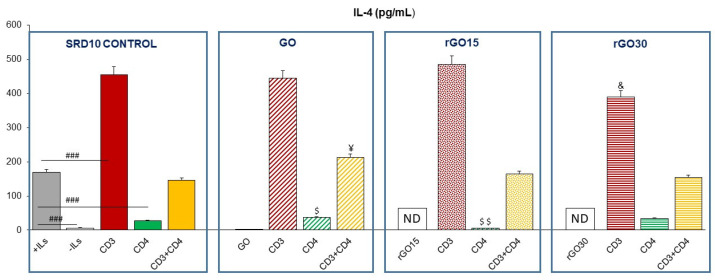
Effect of GO, rGO15 and rGO30 on interleukin-4 (IL-4) secretion by CD4^+^ Th2 SR.D10 cell line after 24 h of treatment with 5 µg/mL of these nanostructures, evaluated by ELISA under basal and stimulated conditions. The control cells were cultured in the presence of stimuli without material (SR.D10 CONTROL) in parallel. Statistical significance: ### *p* < 0.005 (compared to control cells without material cultured in the absence of ILs); ^&^ *p* < 0.05 (compared to control cells activated through CD3 in the absence of ILs); ^$^ *p* < 0.05; ^$$^ *p* < 0.01 (compared to control cells activated through CD4 in the absence of ILs); ^¥^ *p* < 0.05 (compared to control cells activated through CD3 and CD4 in the absence of ILs).

As can be seen in Figure 8, in the absence of these nanostructures, the SR.D10 control cells cultured in the presence of an interleukin-rich culture medium (+ILs, gray bar) secreted higher levels of IL-4 than in the absence of interleukins (-ILs, white bar). This is in agreement with the described effect of ILs on the proliferation of this cell type since it requires the presence of 10 U/mL of mrIL-4 for their correct growth [34].

When the levels of IL-4 in the supernatants of stimulated lymphocytes were analyzed, cells activated through the TCR/CD3 receptor and through TCR/CD3+CD4 showed a significant increase both in the absence of material and in the presence of GO, rGO15 and rGO30, compared to control cells without material cultured in the absence of ILs. This increase is more pronounced when stimulated through TCR/CD3. It has been previously shown that the IL-4 secretion by SR.D10 lymphocytes is induced by stimulation through the TCR-CD3 receptor and through TCR/CD3+CD4, but this effect is not observed after soluble CD4 stimulation [34]. In this context, Criado et al. analyzed the effect of CD4 expression on the activation threshold of mouse T-cells. For this purpose, the authors studied the response to antigen and other T-cell receptor (TCR) ligands in a series of CD4 mutants obtained from clone SR.D10. They found that although antigens can stimulate SR.D10 CD4-mutant cells, they require a higher concentration of antigens or more APCs to produce optimal responses. Furthermore, they showed that activation through TCR/CD3 can be CD4-independent if the activation threshold is sufficiently reduced, for example, in cells such as SR.D10. On the other hand, the authors confirmed the importance of CD4-associated tyrosine kinase activity in early TCR/CD3 signaling in this Th2 cell line since, after TCR/CD3 ligation, tyrosine phosphorylation is detected only on those CD3 chains coprecipitated with CD4 [45,46].

These results indicate that the secretion of this anti-inflammatory regulatory cytokine IL-4 is increased in the case of adequate stimulation of the SR.D10 lymphocytes through their receptor and co-receptor in the absence and presence of these nanostructures. Therefore, this study allows us to conclude that the treatment with GO, rGO15 and rGO30 did not alter the Th2 reparative phenotype, characteristic of this T lymphocyte cell line, contributing to a better understanding of the functional state of lymphocytes after treatment with these nanostructures with potential biomedical applications.

## 3. Materials and Methods

### 3.1. GO, rGO15 and rGO30 Nanostructures Preparation

This study used commercial GO (Graphenea^®^, San Sebastián, Spain). Its source (0.4 wt% aqueous solution) has a monolayer content (measured in 0.05 wt%) above 95% and lateral particle size below 10 µm. GO is made by exfoliating graphite with oxidant and acidic reagents. Other than acidic residues, sulphur and manganese are present in the initial GO solution due to the exfoliation process. Dialysis can reduce the presence of these residues. Therefore, commercial GO dispersion was dialyzed with distilled water for a week. This solution was freeze-dried in a Teslar LyoQuest HT40 freeze-dryer (Beijer Electronics Products AB, Malmoe, Sweden) to produce contaminants-free GO sheets. The GO powder was then heat-treated in a Vacutherm vacuum oven (Thermo Scientific, Karlsruhe, Germany) for 15 (rGO15) and 30 (rGO30) minutes.

### 3.2. Structural and Morphological Characterization of GO, rGO15 and rGO30 Nanostructures

FTIR measurements were performed in a Bruker Optics Tensor 27 spectrometer (Bruker, Karlsruhe, Germany), using 256 scans at a 4 cm^−1^ resolution. The GO samples were mixed with potassium bromate and pressed in a hydraulic press (8 T) to prepare the FTIR pellets.

The zeta potential measurements were performed in a ZetaSizer Nano ZS (green badge) model Zen3500, Malvern, Ltd, Malvern, UK. Before measuring aliquots, the aqueous dispersions of the three GO samples were prepared and thoroughly stirred. Each sample’s pH was determined to be 6.8, the value at which the measurements were made.

Raman measurements were carried out at room temperature with a combined Raman-AFM-SNOM confocal microscope WITec alpha300 RAS^+^ (WITec, Ulm, Germany). A Nd:YAG laser operating at 532 nm (power set at 1 mW to avoid laser-induced heating) was used as the excitation source. The Raman confocal microscope was calibrated by acquiring the spectrum of a silicon wafer (532 nm laser source, 0.5 s, 10 acquisitions, 33 mW laser power). The Gaussian function was fitted to the Raman band at 521 cm^−1^, and an error of 0.08 cm^−1^ was obtained. To acquire the Raman spectra of GO and rGO samples, a drop of an aqueous solution of each GO sample was deposited onto a glass slide and let it dry. Each Raman spectra were acquired 10 times with 5s each acquisition, with a 100× objective. All Raman spectra presented in the manuscript are the average of 5 Raman spectra. The background subtraction and normalization were performed using the WITec control 5.3^+^.

The Raman spectra were multi-fitted using Origin 18 software. The first-order bands were fitted using two Gauss and three Pseudo-Voigt functions, and the second-order band was split into four Lorentz functions [29].

### 3.3. Culture of CD4^+^ Th2 Lymphocyte Cell Line SR.D10 for Treatment with GO, rGO15 and rGO30

SR.D10 [34] is a clone obtained from the murine CD4^+^ Th2 cell line, D10.G4.1 [35], which is specific to the conalbumin fragment 134–146 bound to I–Ak class II major histocompatibility complex molecules. It was maintained in Click’s medium supplemented with 10% heat-inactivated fetal calf serum (FCSi) containing 5 U/mL mouse recombinant interleukin-2 (mrIL-2), 10 U/mL mrIL-4 and 25 pg/mL mrIL-1. After 4 days, the cultured cells were collected, exhaustively washed to eliminate added IL and used for in vitro assays. For exposure to GO and rGO15, and rGO30 nanostructures, SR.D10 lymphocytes were seeded in 96-well culture plates at a density of 2.5 × 10^4^ cells/well in 200 μL of Dulbecco’s Modified Eagle’s Medium (DMEM) supplemented with 10% fetal bovine serum (FBS, Gibco, BRL, United Kingdom), L-glutamine 1 mM (BioWhittaker Europe, Verviers, Belgium), penicillin (200 μg/mL, BioWhittaker Europe, Verviers, Belgium) and streptomycin (200 μg/mL, BioWhittaker Europe, Verviers, Belgium) in the presence of interleukins at 37 °C under 5% CO_2_ atmosphere.

For the activation assays, cells were maintained for 2 h in the same culture medium in the absence of ILs. Then, the cells were treated for 24 h in the absence or presence of 5 μg/mL of GO, rGO15 and rGO30 nanostructures, under basal or stimulated conditions. In vitro cell activation was performed by adding monoclonal antibodies Y-CD3-1 (anti-CD3ε), GK1.5 (anti-CD4), at a final concentration of 5 μg/mL [47], and the combination of the two antibodies to the cultured lymphocytes for 24 h. All these products were supplied by Sigma-Aldrich (St Louis, MO, USA). After these treatments, the media supernatants were collected and kept at −20 °C until use. Lymphocyte proliferation was measured using the Cell Counting Kit-8 (CCK-8) protocol (Sigma-Aldrich, St. Louis, MO, USA). After incubation for 3–4 h under 5% CO_2_ atmosphere at 37 °C, samples of 100 μL were collected into 96-well culture plates (Nunc Brand, Rochester, NY, USA), and absorbance was measured at 450 nm.

### 3.4. Effects of GO, rGO15 and rGO30 on the Intracellular Complexity of CD4^+^ Th2 SR.D10 Lymphocytes Evaluated by Flow Cytometry

The effects of GO, rGO15 and rGO30 on the intracellular complexity of CD4^+^ Th2 SR.D10 lymphocytes after 24 h of treatment were quantified by flow cytometry analyzing 90° light scatter (side scatter, SSC). The SSC parameter is proportional to the intracellular complexity determined in part by the cellular cytoplasm, mitochondria and pinocytic vesicles [36]. The data acquisition and analysis conditions were established using negative and positive controls with the CellQuest Program of Becton Dickinson, and these conditions were maintained in all the experiments.

### 3.5. Cell Viability Studies

Cell viability was measured by adding 0.005% (wt/vol) propidium iodide (PI) in PBS (Sigma-Aldrich, St. Louis, MO, USA) into the samples to stain the dead cells. The PI exclusion indicates the plasma membrane integrity. PI fluorescence was detected in a FACScalibur Becton Dickinson flow cytometer (Becton Dickinson, San Jose, CA, USA) with a 530/30 filter, exciting the sample at 488 nm.

### 3.6. Intracellular Reactive Oxygen Species (ROS) Content of CD4^+^ Th2 SR.D10 Lymphocytes Analyzed by Flow Cytometry

After exposure to the GO, rGO15 and rGO30 nanostructures for 24 h, CD4^+^ Th2 SR.D10 lymphocytes were incubated with 10 μM of 2′,7′-dichlorodihydro fluorescein diacetate (DCF-H2-DA, Serva, Heidelberg, Germany) during 45 min at 37 °C. The non-fluorescent DCF-H2-DA transforms into 2′,7′-dichlorofluorescein (DCF) after hydrolysis by cellular esterases and oxidation by ROS. When DCF is excited at 488 nm emission wavelengths, green fluorescence is emitted that can be detected at 525 nm. DCF fluorescence was measured in a FACScalibur Becton Dickinson flow cytometer with a 530/30 filter, exciting the sample at 488 nm.

### 3.7. Cell-Cycle Phases of CD4^+^ Th2 SR.D10 Lymphocytes Analyzed by Flow Cytometry

The cells in 0.5 mL of PBS were mixed with 4.5 mL of ethanol 70% and maintained overnight at 4 °C. Cell suspensions were then centrifuged for 10 min at 310× *g* and resuspended in 0.5 mL of RNAsa solution containing 0.1% Triton X-100, 20 µg/mL of IP and 0.2 mg/mL of RNAsa (Sigma-Aldrich, St. Louis, MO, USA). After 30 min of incubation at 37 °C, PI fluorescence was detected in a FACScan Becton Dickinson flow cytometer with a 585/42 filter, exciting the sample at 488 nm. The CellQuest Program of Becton Dickinson was used to calculate the percentage of cells in each cycle phase: G0/G1 (growth), S (DNA synthesis) and G2/M (growth and mitosis).

### 3.8. Detection of Interleukin IL-4

The amount of IL-4 secreted by CD4^+^ Th2 SR.D10 lymphocytes under the different conditions was quantified in the culture medium by an enzyme-linked immunosorbent assay (ELISA, Gen-Probe, Diaclone, Besançon, France) according to the manufacturer’s instructions.

### 3.9. Statistics

Data are expressed as means ± standard deviations of a representative of three experiments carried out in triplicate. For statistical significance, at least 10,000 cells were analyzed by flow cytometry in each sample. Statistical analysis was performed using the Statistical Package for the Social Sciences (SPSS) version 22 software. Statistical comparisons were made by analysis of variance (ANOVA). A Scheffé test was used for post-hoc evaluations of differences among groups. In all the statistical evaluations, *p* < 0.05 was considered statistically significant.

## 4. Conclusions

This comparative study with CD4^+^ Th2 SR.D10 lymphocytes treated in vitro with three different graphene-based nanomaterials allowed us to observe the specific response of these cells involved in the adaptive immune response as well as the possible benefits of reducing graphene oxide by vacuum-assisted thermal treatment at 200 °C to improve its biocompatibility and increase its potential for the preparation of scaffolds in tissue engineering.

## Data Availability

Data is contained within the article.

## Supplementary Materials

The following supporting information can be downloaded at: https://www.mdpi.com/article/10.3390/ijms231810625/s1.
