# Supplementary material for: Effects of Graphene Oxide and Reduced Graphene Oxide Nanostructures on CD4+ Th2 Lymphocytes
Source: Int J Mol Sci. 2022 Sep 13;23(18):10625. doi: 10.3390/ijms231810625 (PMC9506555; doi:10.3390/ijms231810625)
Supplement: Supplementary file 1 [file ijms-23-10625-s001.zip › ijms-1851813-supplementary.pdf]

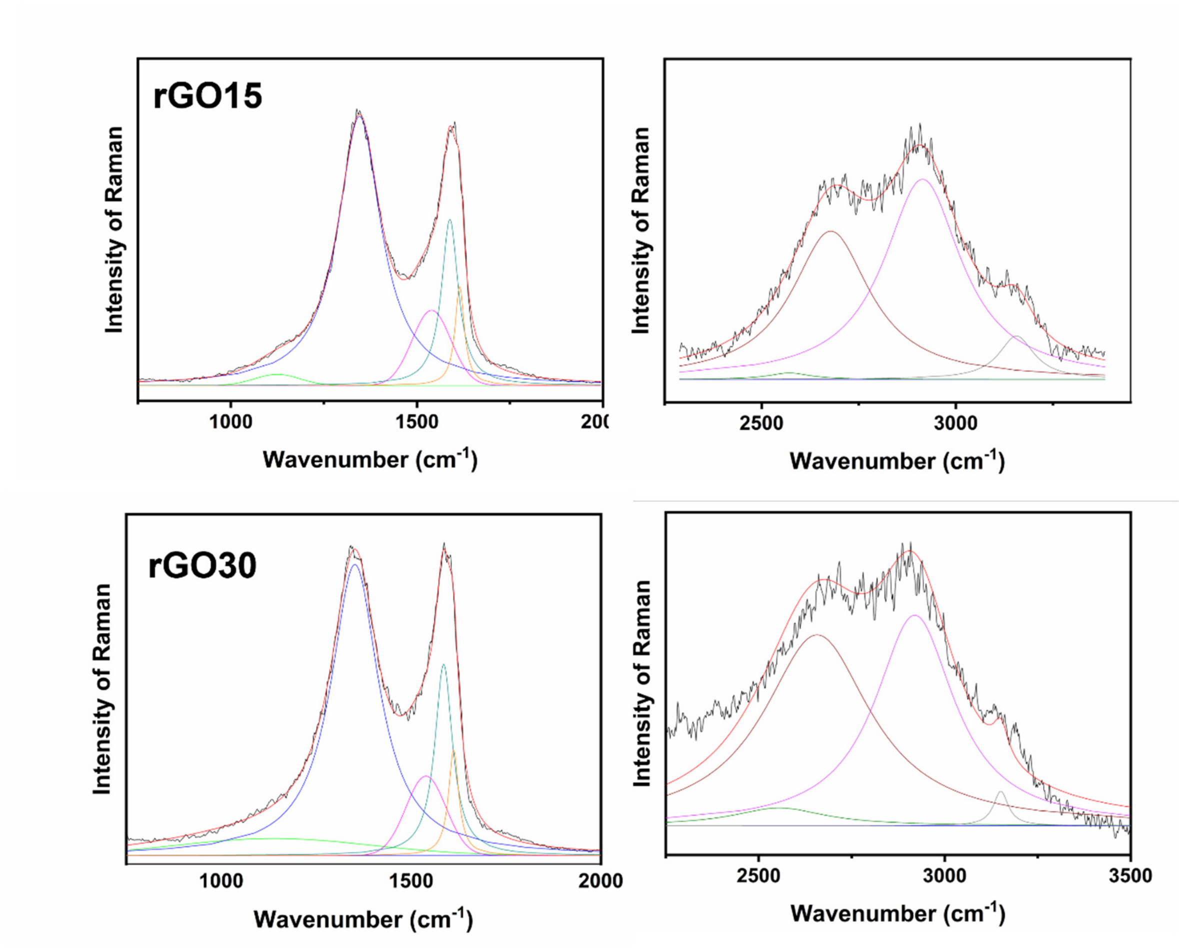

Figure S1: Deconvolution of first and second order Raman spectrum of reduced-graphene oxides

Table S1: Fitting parameters calculated for different rGO in the first and second order Raman spectra.

Material	D* (Gauss)			D (Ps-Voight)			D'' (Gauss)			G (Ps-Voight)			D' (Ps-Voight)		
	xc (cm^{-1})	w (cm^{-1})	A (%)	xc (cm^{-1})	w (cm^{-1})	A (%)	xc (cm^{-1})	w (cm^{-1})	A (%)	xc (cm^{-1})	w (cm^{-1})	A (%)	xc (cm^{-1})	w (cm^{-1})	A (%)
GO	1122	125	1	1347	134	62	1520	114	8	1582	65	20	1611	35	8
rGO 15 min	1125	123	2	1345	141	65	1538	104	12	1588	55	16	1615	30	5
rGO 30 min	1138	512	10	1352	106	60	1539	106	10	1586	57	15	1612	33	5

Material	G* (Lorentz)			2D (Lorentz)			D+D' (Lorentz)			2D' (Lorentz)		
	xc (cm^{-1})	w (cm^{-1})	A (%)	xc (cm^{-1})	w (cm^{-1})	A (%)	xc (cm^{-1})	w (cm^{-1})	A (%)	xc (cm^{-1})	w (cm^{-1})	A (%)
GO	2529	71	0,3	2682	253	42	2920	235	52	3158	105	6
rGO 15 min	2571	130	1	2678	245	40	2914	243	54	3156	110	6
rGO 30 min	2555	300	4	2657	351	50	2919	215	44	3151	48	1

Table S2: Relative intensity and integrated area for some Raman bands of rGO.

Materials	I_D/I_G	$A_{D'}/A_G$	A_D/A_G	$A_{D+D'}/A_D$	$A_{2D'}/A_D$	$A_{2D'}/A_{D+D'}$
GO	1,6	0,4	3,1	0,83	0,09	0,12
rGO 15 min	1,79	0,31	4,06	0,83	0,09	0,11
rGO 30 min	1,72	0,33	4	0,73	0,02	0,02
